# Liraglutide ameliorates beta-cell function, alleviates oxidative stress and inhibits low grade inflammation in young patients with new-onset type 2 diabetes

**DOI:** 10.1186/s13098-018-0392-8

**Published:** 2018-12-17

**Authors:** Wen-qiang Zhang, Yuan Tian, Xiao-min Chen, Li-fen Wang, Chan-chan Chen, Chuan-mei Qiu

**Affiliations:** 1 Department of Endocrinology and Metabolism, Zhongshan Hospital Xiamen University, 201-209 Hubin South Road, Xiamen, 361004 China; 2 Guangzhou Medicine University Second Affiliated Hospital, 250-296 Changgang East Road, Guangzhou, 510260 China

**Keywords:** Liraglutide, Metformin, Type 2 diabetes mellitus, Beta-cell function, Oxidative stress, High sensitivity C-reactive protein

## Abstract

**Background:**

The prevalence of type 2 diabetes in youth is escalating rapidly. We aimed to evaluate the effects of liraglutide on beta-cell function, metabolic productions of oxidative stress, low grade inflammation compared with metformin in young patients with recent onset type 2 diabetes mellitus.

**Methods:**

Sixty patients were randomly assigned to receive 8-week liraglutide or metformin treatment. Beta-cell function was assessed by modified beta cell function index (MBCI), early phase of insulin secretion index (ΔI30/ΔG30), proinsuin to insulin ratio (P/I) and the insulin area under the curve (AUCins). The expression of 8-OH-dG and 8-iso-PGF_2α_ and hs-C-reactive protein (hs-CRP) were measured as indications of oxidative stress and low grade inflammation.

**Results:**

After 8 weeks liraglutide treatment, MBCI, ΔI30/ΔG30, AUCins significantly increased, 8-OH-dG, 8-iso-PGF_2α_, P/I and hs-CRP remarkably reduced. The differences before and after 8-week liraglutide treatment in ΔMBCI (11.1 [2.81, 43.08] vs 0.00 [− 8.16, 10.47], P = 0.017), ΔLNΔI30/ΔG30 (0.44 [0.04, 0.85] vs − 0.09 [− 0.33, 0.36], P = 0.049), ΔAUCins (117 [− 8, 376] vs − 21 [− 314, 109] mIU/L, P = 0.013), ΔP/I (− 0.05 [− 0.09, − 0.03] vs − 0.02 [− 0.04, 0.01], P = 0.026)were remarkably enhanced compared to those of the metformin therapy. The expression of 8-OH-dG, 8-iso-PGF_2α_ and hs-CRP also decreased after 8-week metformin treatment.

**Conclusions:**

These data demonstrated that liraglutide administration was more effective on ameliorating beta-cell function than metformin treatment in young patients with new-onset type 2 diabetes mellitus. Both liraglutide and metformin could alleviate the level of oxidative stress and attenuate low grade inflammatory, we speculate this effect may not the main mechanism of beta-cell function improvement by liraglutide in diabetic patients.

*Trial registration* Chinese Clinical Trials registry, chiCTR1800018008, Registered 27 August 2018—retrospectively registered.

**Electronic supplementary material:**

The online version of this article (10.1186/s13098-018-0392-8) contains supplementary material, which is available to authorized users.

## Background

The latest epidemiological surveys have documented that the prevalence rate of diabetes in adults over 18 years of age in China reaches to 10.9% [1], and exhibited the quickly increasing trend in young patients. Lifestyle changes such as higher fat intake and less physical activity are readily to suffer form T2DM in China, especially to young people. T2DM in east Asians is characterized primarily by beta-cell dysfunction, which is evident immediately after ingestion of glucose or mixed meal, less obesity and younger age of onset compared to Caucasians [2]. Reduced insulin secretory capacity and impaired beta-cell compensation are thought as the two major pathophysiological mechanism of beta-cell dysfunction in type 2 diabetes. At the last decade, incretin has received more and more attentions as a new treatment option for young patients with T2DM, and exerted greater glucose-lowering efficacy in East Asians [3]. Glucagon like peptide-1 (GLP-1) is an incretin hormone produced in the intestinal L cells, which stimulates glucose-dependent endogenous insulin release, decreases glucagon secretion, slows gastric motility and emptying, reduces appetite and food intake [4, 5]. Liraglutide, a long-acting GLP-1 receptor agonists, has been demonstrated that it could improve pancreatic beta cell mass and ameliorate insulin secretion capacity in the animal experiment and large prospective LEAD trial [6, 7]. However, the precise mechanisms behind this benefit effect of liraglutide remain unclear. This study aimed to investigate the effects of liraglutide versus metformin on islet beta-cell function, metabolic products of oxidative stress and C-reactive protein (CRP) in young patients with recent onset type 2 diabetes mellitus.

## Methods

### Subjects

Sixty subjects with type 2 diabetes were enrolled between April 2015 and December 2016 at Xiamen University Affiliated Zhongshan Hospital in China in the department of endocrinology and metabolism and physical examination center. Inclusion criteria for the initial selection were: the patients were initially diagnosed as type 2 diabetes according to World Health Organization criteria, who were 18–40 years of age, had a body mass index (BMI) of 25–35 kg/m^2^, had HbA_1_c between 6.5 and 9%, without therapy for diabetes including diet and exercise, antidiabetes agents prior to study. Exclusion criteria were set as follows: type 1 diabetes, recent acute complications including diabetic ketoacidosis and hyperglycaemic hyperosmolar coma, acute infection, impaired liver function, impaired renal function (creatinine clearance < 45 mL/min) [8], women in pregnancy or lactation, smoker. The study was approved by the ethics committee of Zhongshan Hospital Xiamen University and conducted in according to Helsinki Declaration [9]. Written informed consent was obtained for experimentation with each participant.

### Research design

In this 8-week, randomized, active-control, parallel trial, sixty subjects with type 2 diabetes were randomly assigned (1:1) to receive subcutaneous liraglutide (Novo Nordisk company) or oral metformin (Sino American Shanghai Squibb Pharmaceutical Co.). Metformin were administered at a dose of 1–2 g/day for 8 weeks. Liraglutide started at once-daily dose of 0.6 mg/d for 1 week, increased up to 1.2 mg/day for 7 weeks. Before the study and after 8-week treatment, a 75 g oral glucose tolerance test (OGTT) was conducted for each participant. Blood samples were drawn before and 30, 60, 120 min after OGTT, respectively. At the same time, participant provided a clean-catch 24-h urine sample, which was immediately separated into 1.5 mL aliquots after collection and stored at − 80 °C until analysis. All subjects were given diet and exercise education by professional nurses. There was a follow-up visit once per month. The plasma glucose, body weight, waist circumference, hip circumference and blood pressure were measured and adverse events were monitored during the follow-up period. At the end of the trial, the clinical and laboratory indices were assessed, as previously described.

### Clinical and laboratory measurement

Body weight, height, waist circumference (WC), hip circumference, systolic blood pressure (SBP), diastolic blood pressure (DBP) were collected by professional nurses. The body mass index (BMI) was calculated as the body weight in kilograms divided by the square of the patient’s height in metres. WC was measured midway between the lowest rib and the top of the iliac crest. Hip circumference was measured around the peak of the buttocks. Blood pressure was measured with a mercury manometer on the right arm, after taking rest of 5 min in the sitting position. After an overnight fast, blood samples were drawn for measurements of hemoglobin A_1_c (HbA_1_c), plasma glucose (PG), plasma insulin (INS), lipid profile, proinsulin and hs-CRP. Subsequently, a 75 g oral glucose tolerance test (OGTT) was performed, and plasma glucose and insulin were measured at 0 min and 30 min, 60 min, 120 min after OGTT. PG were measured by the hexokinase method. The plasma glucose, renal and liver functions, plasma lipids and lipoprotein concentrations including triglycerides (TG), total cholesterol (TC), low-density lipoprotein cholesterol (LDL-C), and high-density lipoprotein cholesterol (HDL-C) were performed using an automated method (Roche cobas8000 automatic biochemical analyser). HbA_1_c was measured by HPLC (Bio-Rad, Inc., Hercules, CA, USA). Plasma insulin levels were measured using the electrochemiluminescence immunoassay (ECLI). Proinsuin concentrations were assessed by ELISA kit (Arigo bioaboratories Corporation, Enzyme immunoassay Hsinchu city 300, Taiwan). High-sensitivity C-reactive protein (hsCRP) concentrations were measured using immune turbidimetry.

Urine sample were taken for the determination of 8-hydroxy-2′-deoxy guanosine (8-OH-dG) and 8-isoprostane F_2α_ (8-iso-PGF_2α_). Urinary 8-OH-dG concentrations were assayed using a competitive enzyme-linked immunosorbent assay (ELISA) kit (Japan institute for the control of aging, shizuoka pref. Japan) [10]. Urinary 8-iso-PGF_2α_ concentrations were also assayed using competitive ELISA kit (Northwest life science specialities, LLC, Vancouver, Canada) [11]. The intra-assay and inter-assay coefficients of variation (CV) of the ELISA kits mentioned above were all less than 10%.

The formulas which we assessed beta-cell function were shown as follows:

Modified beta cell function index was calculated as MBCI = (INS0 × GLU0)/(GLU120 + GLU60 − 7). INS0 denotes fasting plasma insulin, GLU0 denotes fasting plasma glucose, GLU60 denotes plasma glucose level at 60 min after glucose load, and GLU120 denotes plasma glucose level at 120 min after glucose load [12].

Insulin area under the curve (AUCins) and glucose area under the curve (AUC_GLU_) during the OGTT were analysed using the trapezoidal method [13].

The early phase insulin secretion index was calculated as (ΔI30/ΔG30) = ([insulin at 30 min] − [fasting insulin])/([glucose at 30 min] − [fasting glucose]) [14].

Proinsuin to insulin ratio was abbreviated as P/I [15].

Deltas (Δ) are presented as the difference before and after treatment, which were suitable for the variables ΔMBCI, ΔAUCins, ΔLNΔI30/ΔG30, ΔP/I and ΔAUC_GLU_.

### Statistical analysis

SPSS packages 21 (SPSS software, IBM Inc., USA) and GraphPad Prism version 5.0 (GraphPad software, Inc., La Jolla, CA, USA) was used for statistical analysis and cartography. Normally distributed data were expressed as mean ± standard deviation (SD). Unpaired t test was used to evaluate the relationship between groups before or after treatment. Paired t test was used to identify differences of baseline and post-treatment in the same group. Non normally distributed data were expressed as median (interquartile rang) and the Mann–Whitney U test or Wilcoxon signed rank test was performed. The Mann–Whitney U test was used to identify differences from baseline with post 8-week treatment for 8-OH-dG, 8-iso-PGF2α, hs-CRP, MBCI, ΔI30/ΔG30 and AUCins between the liraglutide and metformin group. Comparisons of ΔMBCI, ΔLNΔI30/ΔG30, ΔP/I and ΔAUCins after 8-week treatment between liraglutide and metformin group were analyzed using the Mann–Whitney U test. Covariance analysis was performed to determine the associations of Δ AUC_GLU_ with baseline MBCI, LNΔI30/ΔG30, P/I and AUCins, it was also used to evaluate the relationship of ΔMBCI, ΔLNΔI30/ΔG30, ΔP/I and ΔAUCins with baseline levels of HbA_1_c, BMI and waist circumference (WC).

Data with the difference before and after treatment of early phase insulin secretion index (ΔI30/ΔG30) were logarithmically transformed prior to analysis. A two-tailed *P* < 0.05 was considered significant.

## Results

### Comparisons of clinical and laboratory characteristics of the study participants

Baseline characteristics of the study participants between two groups were not statistically significant (*P *> 0.05) (shown in Additional file 1: Table S1).

After 8-week liraglutide treatment, FPG (9.40 ± 2.32 vs 7.33 ± 2.06 mmol/L, *P *= 0.024), 30 min PG (15.43 ± 2.96 vs 11.46 ± 3.61 mmol/L, *P *= 0.003), 60 min PG (18.19 ± 3.60 vs 14.64 ± 3.86 mmol/L, *P *= 0.012), 120 min PG (17.68 ± 4.38 vs 12.16 ± 5.78 mmol/L, *P *= 0.002) significantly decreased. shown in Additional file 2: Table S2). At the same time, HbA_1_c (8.36 ± 0.55 vs 6.85 ± 0.71%, *P *= 0.001), BMI (28.63 ± 3.86 vs 27.67 ± 3.62 kg/m^2^, *P *= 0.001) and waist circumference (92 ± 12 vs 88 ± 11 cm, *P *= 0.001) significantly decreased.

Nevertheless after 8-week metformin treatment, only FPG (8.45 ± 1.57 vs 6.67 ± 1.26 mmol/L, *P *= 0.001) significantly decreased, there were no changes in 30 min PG, 60 min PG and 120 min PG before and after metformin treatment (*P *> 0.05) (shown in Additional file 2: Table S2). Both HbA1c (8.35 ± 0.55 vs 6.53 ± 0.65%, P = 0.001) and waist circumference (88 ± 8 vs 85 ± 8 cm, *P *= 0.002) notably reduced, but there were no changes in BMI (*P *> 0.05).

### Liraglutide treatment ameliorated beta-cell function

**Fig. 1:**
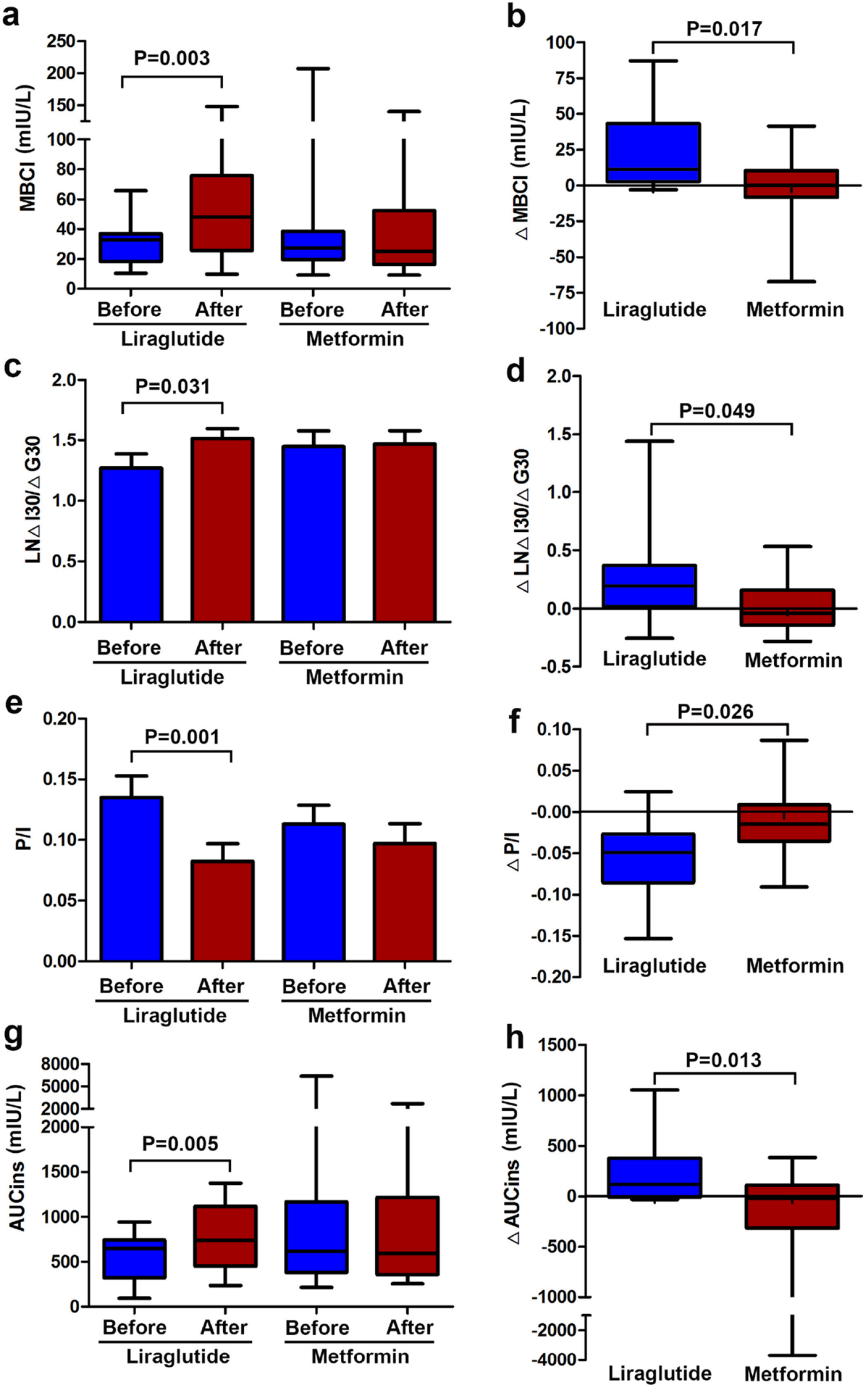
Effect of liraglutide and metformin on beta-cell function. **a** Comparison of modified beta cell function index (MBCI) before and after 8-week treatment. **b** Comparison of the difference of MBCI before and after treatment between two groups. **c** Comparison of log-transformed early phase of insulin secretion (ΔI30/ΔG30) before and after 8-week treatment. **d** Comparison of the difference of LNΔI30/ΔG30 before and after treatment between two groups. **e** Comparison of proinsulin to insulin ratio (P/I) before and after 8-week treatment. **f** Comparison of the difference of P/I before and after treatment between two groups. **g** Comparison of insulin area under the curve (AUCins) before and after 8-week treatment. **h** Comparison of the difference of AUCins before and after treatment between two groups

After 8 weeks liraglutide treatment, MBCI (32.76 [18.23, 36.91] vs 48.01 [25.70, 75.84], *P *= 0.003), ΔI30/ΔG30 (24.94 [7.78, 38.89] vs 31.13 [17.67, 59.09], *P *= 0.031), AUCins (648 [321, 742] vs 738 [451, 1118] mIU/L, *P *= 0.005) significantly increased, The levels of P/I (0.14 ± 0.07 vs 0.08 ± 0.06, *P *= 0.001) were remarkably inhibited (shown in Additional file 2: Table S2 and Fig. 1).

There were no significant changes in MBCI, ΔI30/ΔG30, AUCins and P/I before and after metformin treatment (*P *> 0.05)(shown in Additional file 2: Table S2, Fig. 1).

After 8-week liraglutide treatment, the differences in ΔMBCI (11.1 [2.81, 43.08] vs 0.00 [− 8.16, 10.47], *P *= 0.017), ΔLNΔI30/ΔG30 (0.44 [0.04, 0.85] vs − 0.09 [− 0.33, 0.36], *P *= 0.049), ΔAUCins (117 [− 8, 376] vs − 21 [− 314, 109] mIU/L, *P *= 0.013), ΔP/I (− 0.049 [− 0.086, − 0.027] vs − 0.015 [− 0.036, 0.009], *P *= 0.026) were remarkably enhanced compared to those of the metformin therapy (shown in Table 1 and Fig. 1). However, which were not significant with baseline levels of HbA_1_c, BMI and waist circumference (P > 0.05) (shown in Additional file 3: Table S3).

**Table 1:** Comparisons of ΔMBCI, ΔLNΔI30/ΔG30, ΔP/I and ΔAUCins levels after 8-week treatment between liraglutide and metformin group

Variable	Liraglutide	Metformin	Difference	*P* value
ΔMBCI	11.1 (2.81, 43.08)	0.00 (− 8.16, 10.47)	6.53 (− 1.75, 14.02)	0.017
ΔLNΔI30/ΔG30	0.44 (0.04, 0.85)	− 0.09 (− 0.33, 0.36)	0.21 (− 0.15, 0.68)	0.049
ΔP/I	− 0.05 (− 0.09, − 0.03)	− 0.02 (− 0.04, − 0.01)	− 0.03 (− 0.06, − 0.01)	0.026
ΔAUCins (mIU/L)	117 (− 8, 376)	− 21 (− 341, 109)	39 (− 33, 227)	0.013

Data are expressed as median (interquartile rang). Deltas (Δ) are presented as the difference of variables before and after treatment

MBCI, modified B cell function index; ΔI30/ΔG30, [(insulin at 30 min) − (insulin at 0 min)]/[(glucose at 30 min) − (glucose at 0 min)]; P/I, proinsuin to insulin ratio; AUCins, insulin area under the curve; LN, log-transformed

In covariance analysis model, the reductions of AUC_GLU_ (ΔAUC_GLU_) after liraglutide and metformin treatment were associated with the baseline MBCI (F = 8.041, P = 0.009), P/I (F = 12.72, P = 0.001), AUCins (F = 14.923, P = 0.001), and LNΔI30/ΔG30 (F = 6.080, P = 0.020) (shown in Table 2).

**Table 2:** Covariate analysis in ΔAUC_GLU_ with baseline MBCI, P/I, AUCins and LNΔI30/ΔG30

Variables	Test statistics with ΔAUC_GLU_ (*F*-value)	*P*-value
MBCI	8.041	0.009
P/I	12.72	0.001
AUCins (mIU/L)	14.923	0.001
LNΔI30/ΔG30	6.080	0.020

Deltas (Δ) are presented as the difference of variables before and after treatment

AUC_GLU_, glucose area under the curve; MBCI, modified B cell function index; P/I, proinsulin to insulin ratio; AUCins, insulin area under the curve; ΔI30/ΔG30, [(insulin at 30 min) − (insulin at 0 min)]/[(glucose at 30 min) − (glucose at 0 min)]; LN, log-transformed

### Liraglutide and metformin treatment inhibited oxidative stress and low grade inflammatory

The levels of 8-OH-dG (35.95 [29.30, 50.70] vs 18.74 [4.84, 24.20]ng/mL, *P *= 0.002), 8-iso-PGF_2α_ (1345 [885, 1920] vs 288 [183, 472]ng/mL, *P *= 0.001), hs-CRP (1.96 [1.11, 3.89] vs 1.47 [0.53, 1.86]mg/L, *P *= 0.002)were remarkably inhibited after 8-week liraglutide treatment (shown in Table 3). The expression of 8-OH-dG (16.77 [9.71, 32.60] vs 7.86 [2.87, 23.31]ng/mL, *P *= 0.027), 8-iso-PGF_2α_ (1180 [1025, 1765] vs 299 [228, 586] ng/mL, *P *= 0.001) and hs-CRP (1.88 [1.06, 3.69] vs 1.44 [0.67, 2.35] mg/L, *P *= 0.017) also decreased after 8-week metformin treatment (shown in Table 3).

**Table 3:** Comparisons the levels of 8-OH-dG, 8-iso-PGF2α and hsCRP before and after 8-week treatment between two groups

Variable	Liraglutide group			Metformin group		
	Pre-treatment	Post-treatment	P-value	Pre-treatment	Post-treatment	P-value
8-OH-dG (ng/mL)	35.95 (29.30, 50.70)	18.74 (4.84, 24.20)	0.002	16.77 (9.71, 32.60)	7.86 (2.87, 23.31)	0.027
8-iso-PGF2α (ng/mL)	1345 (885, 1920)	288 (183, 472)	0.001	1180 (1025, 1765)	299 (228, 586)	0.001
hsCRP (mg/L)	1.96 (1.11, 3.89)	1.47 (0.53, 1.86)	0.002	1.88 (1.06, 3.69)	1.44 (0.67, 2.35)	0.017

Data are expressed as median (interquartile rang)

8-OH-dG, 8-hydroxy-2′-deoxyguanosine; 8-iso-PGF_2_α, 8-isoprostane F_2_α; hsCRP, high sensitivity C-reactive protein

## Discussion

Our data show the human GLP-1 analogue liraglutide ameliorates beta-cell function and insulin secretion capacity compared with 8-week metformin treatment in young patients with new-onset type 2 diabetes mellitus. In this study, we combined the modified beta cell function index (MBCI), early phase of insulin secretion (ΔI30/ΔG30), fasting proinsulin to insulin ratio (P/I) with the insulin area under the curve (AUCins) to assess the beta-cell function and insulin secretion capacity. We found the levels of MBCI, ΔI30/ΔG30, AUCins increased by 47%, 25% and 14% respectively, the ratio of P/I remarkably reduced 43% compared with baseline after 8-week liraglutide treatment. However, no statistical changes of MBCI, P/I, ΔI30/ΔG30 and AUCins were achieved in the metformin treatment group.

As we known, type 2 diabetes (T2D) is a progressive disease characterized by both beta-cell deficit and insulin resistance. Previous reports have shown that beta-cell volume decreased by 63% in obese T2DM patients due to increasing threefold beta-cell apoptosis [16], which suggested that improvement of beta-cell dysfunction may be an important therapeutic strategy for the treatment of T2DM. GLP-1 is an incretin hormone secreted by intestinal epithelial L cells that promotes glucose-dependent insulin secretion, decreases glucagon secretion, stimulates beta-cell proliferation, suppresses apoptosis, and restores the function of islet beta-cells [17–19]. It is widely recognized that T2DM in East Asians is characterized primarily by beta-cell dysfunction, which is evident immediately after ingestion of glucose or meal, and less adiposity compared to the disease in Caucasians [20]. Interestingly, the glucose-lowering efficacy of glucagon-like peptide-1 receptor agonists was reported to be greater in Asians than in non-Asians. The difference in the GLP-1 treatment responses could be ascribed to a different pathophysiology of type 2 diabetes, namely, lower insulin secretory function and less insulin resistance, lower body mass index, different genetic makeups, preserved incretin effect and different food compositions in East Asians compared with other ethnic groups [21]. We have also documented that the reductions of AUC_GLU_ (ΔAUC_GLU_) after liraglutide or metformin treatment were associated with the baseline MBCI, P/I, AUCins, and LNΔI30/ΔG30 by covariance analysis, in other words, HbA1c-lowering effects of liraglutide depends on remaining beta-cell function.

Liraglutide protected against reductions of beta-cells in a glucokinase–independent fashion and increased glucokinase protein expression, which was correlated to beta-cell threshold sensitivity to glucose [22]. Liraglutide also improved the proliferation and insulin secretion of beta-cell in high FFAs condition, which enhanced pancreatic and duodenal homeobox 1 (PDX-1) and MafA and NeuroD expressions, down-regulated of p27, Bax expressions, induced the phosphorylation of FoxO1 by activation of PI3K/Akt signalling pathway [23].

Degn et al. reported that beta-cell function in the fasting state, as assessed by HOMA-B analysis, was increased by 30%, first-phase insulin response after the intravenous glucose bolus was increased by 60% after 1 week of liraglutide administration. The proinsulin/insulin ratio was reduced by 40–50%, mean insulin concentration was increased by 2- to 3.5- fold, mean circulating glucagon concentration was reduced by 20% during the hyper-glycemic clamp. Our findings are generally consistent with previous literature [24], document that liraglutide efficiently improves beta-cell function and insulin secretion capacity, which were not correlated with baseline levels of HbA_1_c, BMI and waist circumference. Our results suggest that improvement of beta-cell function was independent of the basal values on glucose and weight.

We demonstrated in this study that liraglutide and metformin treatment significantly reduced the expression of urinary 8-OH-DG and 8-iso-PGF_2α_.than those of baseline. At the same time, we also demonstrated that liraglutide treatment inhibited the expression of sVCAM-1 and hs-CRP [25]. 8-Hydroxy-2′-deoxyguanosine (8-OHdG), produced by oxidation of the nucleoside deoxyguanosine and subsequently excreted directly into urine, has been considered as a sensitive marker for oxidative DNA damage [26]. 8-iso-PGF_2α_ derived from arachidonic acid, which was formed non-enzymatically through oxygen radicals, induced peroxidation of membrane phospholipids [27]. Urinary 8-OH-dG and 8-iso-PGF_2α_ levels have been validated as sensitive biomarkers of oxidative stress in large-scale human studies [28].

Increased levels of oxidative stress exerted deleterious effect on beta-cell function, impaired glucose tolerance and ultimately leading to T2DM. Beta cells are particularly sensitive to ROS because there are relatively low levels of antioxidant enzymes, then oxidative stress should damage mitochondria and markedly blunt insulin secretion, specifically for early phase of insulin secretion [29, 30]. Oxidative stress impaired insulin action through an increase in intracellular calcium concentration or a reduction in nitric oxide availability [31, 32].

However, the precise mechanisms behind the effects of liraglutide on the signalling pathways that attenuate oxidative stress and anti-inflammation are not fully elucidated, although several hypotheses have been proposed. First, in diabetic db/db mice, liraglutide treatment for 2 weeks significantly increased the expression of genes involved in anti-oxidative stress (Cat and Gpx) and reduced endoplasmic reticulum stress in beta-cells, by binding with GLP-1 receptors, which activates adenylate cyclase and the cyclic AMP/protein kinase A (PKA) signalling pathway. Liraglutide also activates phosphoinositide 3-kinase (PI3K), p42 mitogen-activated protein kinase (MAPK) and the epidermal growth factor receptor [33]. Second, liraglutide time-dependent increased phosphorylation of the pro-survival kinase AKT, which was completely inhibited by the PI3K inhibitor wortmannin, demonstrated that phosphorylation of AKT was PI3K dependent [34]. Third, on a rat stroke model, wistar rats received occlusion of the middle cerebral artery for 90 min, liraglutide or saline was administered intraperitoneally at 1 h after reperfusion, liraglutide-treatment significantly reduced the level of derivatives of reactive oxygen metabolites (d-ROMs), compared with that of control, which demonstrated administration of GLP-1 suppressed glucose-stimulated inducible nitric oxide synthase (iNOS) activity and expression and its stimulation of insulin release in pancreatic islet cells at least partly through PKA signalling [35, 36]. Fourth, upon TNF-α-induced injury of the human umbilical vein endothelial cells (HUVECs), liraglutide inhibited rapid translocation of PKC-α into membrane, inhibited NF-κB signaling activation and NADPH oxidase, inhibited apoptosis of HUVEC and expression of Pentraxin-3, increased the levels of SOD-2, catalase and GPx, liraglutide exerts marked anti-oxidative and anti-inflammatory effects [37].

The strengths of the current study include the randomized, active controlled design and consistent baseline with few interference factors. To the best of our knowledge, this is the first study to combine four indexes with MBCI, P/I, Δ I30/Δ G30 and AUCins at the same time to evaluate the protective effects of liraglutide on beta-cell function. In addition, we collected 24 h of urine, not random urine tests, to assess the levels of urinary 8-OH-dG and 8-iso-PGF2α, which was more reliable to confirm the anti-oxidative capacity. Despite of our efforts to plan and complete the whole research, there are still some limitations. First, compared with the large longitudinal study, it has a non-blinded design, lacks a blank control group, has relatively small sample size and comparatively short study period. Second, further studies are needed to reveal the relevant signalling pathways by which liraglutide exerts beneficial influence on islet beta-cell function against oxidative stress and inflammation.

## Conclusions

our findings indicate liraglutide administration was more effective on ameliorating beta-cell function than metformin treatment in young patients with new-onset type 2 diabetes mellitus. Both liraglutide and metformin could reduce the level of oxidative stress and attenuate low grade inflammatory, we speculate this effect may not the main mechanism of beta-cell function improvement by liraglutide in diabetic patients.

## Additional files

**Additional file 1: Table S1.** Baseline characteristics of the study participants.

**Additional file 2: Table S2.** Comparisons of plasma glucose and insulin secretion capacity before and after 8-week treatment between two groups.

**Additional file 3: Table S3.** Covariate analysis on the changes of beta-cell function with baseline HbA_1_c, BMI and WC.

## Abbreviations

T2DM: type 2 diabetes mellitus
BMI: body mass index
WC: waist circumference
TC: total cholesterol
TG: triglycerides
HDL-C: high-density lipoprotein cholesterol
LDL-C: low-density lipoprotein cholesterol
HbA_1_c: glycated haemoglobin
SBP: systolic blood pressure
DBP: diastolic blood pressure
FPG: fasting plasma glucose
FINS: fasting insulin
AUCins: insulin area under the curve
MBCI: modified B cell function index
ΔI30/ΔG30: [(insulin at 30 min) − (insulin at 0 min)]/[(glucose at 30 min) − (glucose at 0 min)]
P/I: proinsuin to insulin ratio
hsCRP: high sensitivity C-reactive protein
8-OH-dG: 8-hydroxy-2′-deoxyguanosine
8-iso-PGF_2_α: 8-isoprostane F_2_α
LN: log-transformed
OGTT: oral glucose tolerance test
GLP-1: glucagon-like peptide-1
LRG: liraglutide
MET: metformin
sVCAM-1: soluble vascular cell adhesion molecule-1
ELISA: enzyme-linked immunosorbent assay
HPLC: high performance liquid chromatography
ECLI: electrochemiluminescence immunoassay
CV: coefficients of variation
SD: standard deviation
PDX-1: pancreatic and duodenal homeobox 1
ROS: reactive oxygen species
AMP: activated protein kinase
AMPK: adenosine monophosphate activated protein kinase
PKA: protein kinase A
PI3K: phosphoinositide 3-kinase
d-ROMs: derivatives of reactive oxygen metabolites
iNOS: inducible nitric oxide synthase
TNF-α: tumor necrosis factor-α
HUVEC: human umbilical vein endothelial cells
MAPK: mitogen-activated protein kinase
NF-kB: nuclear factor kappa-light-chain-enhancer of activated B cells
