# Supplementary material for: Liraglutide ameliorates beta-cell function, alleviates oxidative stress and inhibits low grade inflammation in young patients with new-onset type 2 diabetes
Source: Diabetol Metab Syndr. 2018 Dec 17;10:91. doi: 10.1186/s13098-018-0392-8 (PMC6296090; doi:10.1186/s13098-018-0392-8)
Supplement: Supplementary file 1 — Additional file 1: Table S1. Baseline characteristics of the study participants. [file 13098_2018_392_MOESM1_ESM.doc]

**Table S1 Baseline characteristics of the study participants**

| **Variable** | **Liraglutide**  **(n=30)** | **Metformin**  **(n=30)** | ***P*- value** |
| --- | --- | --- | --- |
| Age (years) | 31.67±5.61 | 33.57±4.99 | 0.171 |
| Male/Female(n) | 20/10 | 20/10 | 1.000 |
| BW(kg) | 81±17 | 72±11 | 0.104 |
| BMI (kg/m^2^) | 28.63±3.86 | 26.16±3.1 | 0.063 |
| Waist Circumference(cm) | 92±12 | 88.±8 | 0.3 0.304 |
| Hip Circumference(cm) | 100±6 | 96±6 | 0.112 |
| SBP (mmHg) | 122± 16 | 120± 11 | 0.643 |
| DBP (mmHg) | 79± 12 | 81± 7 | 0.719 |
| TC(mmol/L) | 4.79±0.87 | 5.02±0.79 | 0.444 |
| TG(mmol/L) | 1.82±0.74 | 1.99±0.74 | 0.539 |
| LDL-C(mmol/L) | 3.61±0.82 | 3.78±0.68 | 0.535 |
| HDL-C(mmol/L) | 1.12±0.29 | 1.20±0.26 | 0.448 |
| HbA1c(%) | 8.36±0.55 | 8.35±0.55 | 0.925 |
| FPG(mmol/L) | 9.40±2.32 | 8.45±1.57 | 0.198 |
| FINS(mIU/L) | 104(51,123) | 76(60, 150) | 0.724 |
| AUCins(mIU/L) | 648(321, 742) | 615(381, 1167) | 0.520 |
| ΔI30/ΔG30 | 24.94(7.78, 38.89) | 30.18(10.4, 53.75) | 0.272 |
| MBCI | 32.76(18.23, 36.91) | 27.36(19.64, 38.60) | 0.806 |
| P/I | 0.14±0.07 | 0.11±0.06 | 0.624 |
| hsCRP (mg/ L) | 1.96(1.11, 3.89) | 1.47（0.53,1.86） | 0.917 |
| 8-OH-dG (ng/ml) | 35.95(29.30, 50.70) | 16.77(9.71, 32.60) | 0.041 |
| 8-iso-PGF_2_α (ng/ml) | 1345(885, 1920) | 1180(1025, 1765) | 0.925 |

Data are expressed as mean ±standard deviation or median( interquartile rang).

BW:body weight; BMI: body mass index; SBP:Systolic blood pressure; DBP: Diatolic blood pressure; LDL-C: low-density lipoprotein cholesterol; HDL-C: high-density lipoprotein cholesterol; HbA_1_c:glycated haemoglobin; FPG:fasting plasma glucose; FINS:fasting insulin: AUCins: insulin area under the curve; MBCI: modified B cell function index; ΔI30/ΔG30: = [(insulin at 30 min) - (insulin at 0 min)]/[(glucose at 30 min) - (glucose at 0 min)]; P/I: proinsuin to insulin ratio; hsCRP:high sensitivity C-reactive protein; 8-OH-dG: 8-hydroxy-2’-deoxyguanosine ;8-iso-PGF_2_α: 8-isoprostane F2α.
