# Supplementary material for: Liraglutide ameliorates beta-cell function, alleviates oxidative stress and inhibits low grade inflammation in young patients with new-onset type 2 diabetes
Source: Diabetol Metab Syndr. 2018 Dec 17;10:91. doi: 10.1186/s13098-018-0392-8 (PMC6296090; doi:10.1186/s13098-018-0392-8)
Supplement: Supplementary file 2 — Additional file 2: Table S2. Comparisons of plasma glucose and insulin secretion capacity before and after 8-week treatment between two groups. [file 13098_2018_392_MOESM2_ESM.doc]

**Table S2. Comparisons of plasma glucose and insulin secretion capacity before and after 8-week treatment between two groups**

| **Variable** | **Liraglutide Group** | | | **Metformin Group** | | |
| --- | --- | --- | --- | --- | --- | --- |
|  | Pre-  treatment | P Post-  treatment | P -value | Pre-  treatment | P Post-  treatment | P  -value |
| FPG  (mmol/L) | 9.40±2.32 | 7.33±2.06 | 0.024 | 8.45±1.57 | 6.67±1.26 | 0.001 |
| 30minPG  (mmol/L) | 15.43±2.96 | 11.46±3.61 | 0.003 | 13.03±2.72 | 12.13±2.07 | 0.078 |
| 60minPG  (mmol/L) | 18.19±3.60 | 14.64±3.86 | 0.012 | 16.43±4.13 | 15.38±3.63 | 0.193 |
| 120minPG  (mmol/L) | 17.68±4.38 | 12.16±5.78 | 0.002 | 14.47±5.05 | 12.43±5.34 | 0.081 |
| FINS  (mIU/L) | 104  (51,123) | 113  (54,171) | 0.015 | 76  (60, 150) | 87  (41, 175) | 0.650 |
| 30min insulin  (mIU/L) | 271  (136, 298) | 291  (173, 333) | 0.307 | 211  (118, 444) | 217  (158, 387) | 0.865 |
| 60min insulin  (mIU/L) | 326  (165, 441) | 471  (334, 717) | 0.005 | 368  (242, 731) | 396  (277, 918) | 0.932 |
| 120min insulin  (mIU/L) | 401  (193, 560) | 500  (367, 960) | 0.047 | 475  (286, 1060) | 542  (208, 866) | 0.460 |
| MBCI | 32.76  (18.23, 36.91) | 48.01  (25.70, 75.84) | 0.003 | 27.36  (19.64,38.60) | 25  (16.18,52.34) | 0.975 |
| ΔI30/ΔG30 | 24.94  (7.78, 38.89) | 31.13  (17.67, 59.09) | 0.031 | 30.18  (10.4,53.75) | 21.96  (14.42,40.93) | 0.650 |
| P/I | 0.14±0.07 | 0.08±0.06 | 0.001 | 0.11±0.06 | 0.10±0.06 | 0.169 |
| AUCins  (mIU/L) | 648  (321, 742) | 738  (451, 1118) | 0.005 | 615  (381, 1167) | 594  (357, 1216) | 0.460 |

Data are expressed as mean ±standard deviation or median( interquartile rang).

FPG:fasting plasma glucose; FINS: fasting insulin; MBCI: modified B cell function index; ΔI30/ΔG30= [(insulin at 30 min) - (insulin at 0 min)]/[(glucose at 30 min) - (glucose at 0 min)];

P/I: proinsuin to insulin ratio; AUCins: insulin area under the curve;
