# Supplementary material for: Liraglutide ameliorates beta-cell function, alleviates oxidative stress and inhibits low grade inflammation in young patients with new-onset type 2 diabetes
Source: Diabetol Metab Syndr. 2018 Dec 17;10:91. doi: 10.1186/s13098-018-0392-8 (PMC6296090; doi:10.1186/s13098-018-0392-8)
Supplement: Supplementary file 3 — Additional file 3: Table S3. Covariate analysis on the changes of beta-cell function with baseline HbA1c, BMI and WC. [file 13098_2018_392_MOESM3_ESM.doc]

**Table S3 Covariate analysis on the changes of beta-cell function with baseline HbA_1_c, BMI and WC**

| **Variable** |  | ΔMBCI | ΔLNΔI30/ΔG30 | ΔP/I | ΔAUCins |
| --- | --- | --- | --- | --- | --- |
| HbA_1_c  (%) | ***F*** | 0.167 | 0.300 | 0.039 | 0.310 |
|  | ***P*** | 0.686 | 0.589 | 0.845 | 0.582 |
| BMI (kg/m^2^) | ***F*** | 0.008 | 0.150 | 0.018 | 6.781 |
|  | ***P*** | 0.929 | 0.701 | 0.896 | 0.015 |
| WC  (cm) | ***F*** | 0.264 | 0.259 | 1.044 | 4.115 |
|  | ***P*** | 0.611 | 0.615 | 0.316 | 0.052 |

Deltas(Δ) are presented as the difference of variables before and after treatment

MBCI : modified B cell function index; P/I : : proinsuin to insulin ratio; AUCins: insulin area under the curve; ΔI30/ΔG30= [(insulin at 30 min) - (insulin at 0 min)]/[(glucose at 30 min) - (glucose at 0 min)]; HbA_1_c:glycated haemoglobin; BMI: body mass index; WC:waist Circumference.;LN:log-transformed .
